# Poly(hydroxyalkanoate)s-Based Hydrophobic Coatings for the Protection of Stone in Cultural Heritage

**DOI:** 10.3390/ma11010165

**Published:** 2018-01-20

**Authors:** Serena Andreotti, Elisa Franzoni, Paola Fabbri

**Affiliations:** 1 Department of Civil, Chemical, Environmental and Materials Engineering, University of Bologna, 40131 Bologna, Italy; serena.andreotti2@unibo.it (S.A.); p.fabbri@unibo.it (P.F.); 2 Consorzio Interuniversitario di Scienza e Tecnologia dei Materiali (INSTM), 50121 Firenze, Italy

**Keywords:** protectives, water repellency, bio-based biodegradable polymers, conservation, PHA, marble, limestone, sandstone

## Abstract

Reversibility is a mandatory requirement for materials used in heritage conservation, including hydrophobic protectives. Nevertheless, current protectives for stone are not actually reversible as they remain on the surfaces for a long time after their hydrophobicity is lost and can hardly be removed. Ineffective and aged coatings may jeopardise the stone re-treatability and further conservation interventions. This paper aims at investigating the performance of PHAs-based coatings for stone protection, their main potential being the ‘reversibility by biodegradation’ once water repellency ended. The biopolymer coatings were applied to three different kinds of stone, representative of lithotypes used in historic architecture: sandstone, limestone and marble. Spray, poultice and dip-coating were tested as coating techniques. The effectiveness and compatibility of the protectives were evaluated in terms of capillary water absorption, static and dynamic contact angles, water vapour diffusion, colour alteration and surface morphology. The stones’ wettability after application of two commercial protectives was investigated too, for comparison. Finally, samples were subjected to artificial ageing to investigate their solar light stability. Promising results in terms of efficacy and compatibility were obtained, although the PHAs-based formulations developed here still need improvement for increased durability and on-site applicability.

## 1. Introduction

The protection of architectural elements against water is one of the main challenges for the conservation of cultural heritage, as water is a major cause of material degradation, which may be physical, mechanical or chemical [1,2,3,4,5,6,7,8]. In this context, water may have different origins, such as rain, relative humidity (condensation), or capillary rise from soil. Protection requires an accurate analysis of the water source and paths and a subsequent design of drain systems to control the water run-off from the top to the bottom of the construction. However, this is often not possible or not sufficient in heritage buildings, due to several existing restraints. For this reason, an approach combining chemical and physical protection is often needed to mitigate the problem, and the application of hydrophobic coatings over the exposed surface, preventing water penetration through the material porosity without hindering water vapour transport, has been demonstrated to be an effective solution [4,9,10,11]. 

The most common organic-based hydrophobic protectives that are currently available, such as silanes and siloxanes, waxes, acrylic resins or fluorinated polymers, have shown some limitations [5], such as short-term water repellency [12] due to polymer ageing [1], or undesired effects such as yellowing or coating detachment (due to poor compatibility with inorganic substrates [3,4,13]). Many studies were targeted to improve organic-based formulations by means of inorganic nanoparticle addition [3,4,10,13,14,15], for increasing the coating hydrophobicity [3,4,10,14] or providing the surface with self-cleaning properties [10,15,16,17]. A different approach, based on inorganic treatments, was proposed by other authors [12,13,18], mostly for the protection of calcitic materials from acid or clean rain, hence specifically targeted to preventing stone dissolution rather than hindering water ingress into stone. These treatments provide good compatibility and durability [14], but may exhibit limited penetration into the substrate [3] or the formation of cracks, which limit their protective performance [13]. 

However, in the development of protective formulations, besides achieving good performance, the requirements imposed by the Restoration Charters [19,20] must be fulfilled. In fact, any protective coating should be not only effective, but also compatible with the substrate (without altering the water vapour permeability and aesthetic appearance—such as colour and reflectance—too much, or causing unwanted damage) and durable, but also reversible, i.e., removable at some future date, should that prove necessary [3,8,9,21]. However, in the context of stone conservation, reversibility is “more idealistic than realistic” [21], as even the most soluble of treatments can be extremely difficult to remove. For this reason, any treatment is required to be at least retreatable. Reversibility and retreatability are becoming more and more crucial in conservation, because the number of interventions in buildings already restored in the past is increasing. Notably, incompatible or simply ineffective protectives that cannot be removed from the substrate may jeopardise the subsequent repair treatments. Organic protectives, although they were considered reversible in the 1970s and 1980s by means of suitable solvent application, are actually very hard to remove after decades of exposure to outdoor conditions, which irreversibly altered their composition and properties [22,23]. Hence, after losing their water repellency effectiveness (after 5–10 years according to some authors [24,25]), these protectives remain on the surface of building materials for a long time, subject to continuous ageing. As a result, the dictum of reversibility and retreatability of water repellents represents a great challenge for the scientific community, as it is necessary to ensure that there are no unforeseen consequences of multiple applications of maintenance coatings [21].

In recent years, biopolymers have attracted interest as protective materials for stones [26,27], due to their potential compliance with the reversibility/retreatability requirement. Although biodegradable polymers cannot be properly defined as ‘reversible’ (they do not exhibit any improved solubility with respect to current protectives) or ‘retreatable’ (they do not exhibit any particular compatibility with new coatings of a different nature), they are expected to completely disappear from the stone once their water repellency action has ceased, without jeopardising or influencing further treatments. For this reason, biopolymers might be considered ‘intrinsically reversible’, as they do not leave any permanent residue in the stone and do not cause any unforeseen consequences in subsequent conservation work.

So far, zein, chitosan, poly(3-hydroxybutyrate) (PHB) and poly(lactic acid) (PLA) have been tested as marble coatings against sulphation [25], although the results are only preliminary. PLA has been studied in association with fluorine, by means of the synthesis of fluorinated PLA copolymers [27,28] with nanoparticles [29] or with both fluorine and nanoparticles [30] to enhance the water repellency of the coatings, but the evaluation of their effectiveness is still at a preliminary stage. 

Poly(hydroxyalkanoate)s (PHAs) are a class of naturally occurring thermoplastic linear polyesters that are synthesised as high molecular weight polymer chains by several species of bacterial strains [31,32,33,34], fed with renewable carbon sources such as sugars and agricultural wastes. PHAs have been well known since the beginning of the last century, but only recently have their peculiar features been taken into consideration for the development of functional and advanced solutions for different fields. At present, PHAs can be used in many applications such as medical implant materials [33,34], drug delivery carriers [31,34], packaging [31,33,34], moulded goods [33], paper coatings [33] and non-woven fabrics [33]. Poly(3-hydroxybutyrate) (PHB) is the most widespread polymer in the PHAs class. It is a highly crystalline linear homopolymer with chemical structure –[O–CH(CH_3_)–CH_2_–(C=O)]_n_–. The physical and mechanical properties of PHB are similar to those of polypropylene [30,32,34], even if PHB is less ductile [31,33,35].

Poly(3-hydroxybutyrate-co-3-hydroxyvalerate-co-4-hydroxyvalerate) (PHBVV) is a statistical copolymer composed of 3-hydroxybutyrate, 3-hydroxyvalerate and 4-hydroxyvalerate repetitive units. Due to its structure and to the presence of hydroxyvalerate moieties, PHBVV is less crystalline, more flexible and tougher than PHB. 

The application of PHAs for the protection of stones in cultural heritage is strongly supported by several peculiarities of these biopolymers: intrinsic hydrophobic nature, which avoids the impellent need to include fluorine and inorganic nanofillers in the protective coating; low acidity, which avoids unfavourable interactions with the stone surface; and biodegradability in environmental conditions, which provides the surface treatment with an intrinsic reversibility (after tailoring the surface treatment duration) once the water-repellent action finishes. 

**Figure 1:**
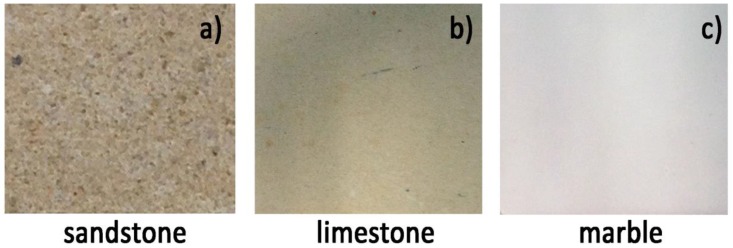
Substrates selected: (**a**) sandstone (Siena stone); (**b**) limestone (Lecce stone); (**c**) marble (Carrara marble).

On this basis, the present research is aimed at developing formulations based on PHAs to be used as protective treatments and evaluating their performance on three different types of stones, namely limestone, sandstone and marble (Figure 1). These stones were selected in order to investigate the protective effectiveness of the treatments on substrates differing in colour, microstructure and chemical composition. PHB and PHBVV solutions were coated onto the stones by different techniques (i.e., spray, poultice and dip coating), as the application method is known to have a strong effect on the amount of material penetrating in the stone or deposited on the surface and in order to assess the influence of the application method onto the overall performance of the treatments. Spray represents the most widely used method for the application of protectives on-site and poultice is an application method commonly used in conservation practice, for both cleaning and consolidation; dip coating, despite being basically not applicable on-site, was adopted to produce a uniform deposition of the protectives on the sample’s surface, hence to investigate the behaviour of the treatment in ‘ideal’ application conditions.

The performance and compatibility of the protective hydrophobic treatments were investigated in terms of capillary water absorption, static and dynamic contact angles, surface tension, water vapour diffusion, colour alteration and surface morphology. 

The stone wettability after the application of the biopolymers was compared with that achieved using two commercial water-repellents widely used for stone conservation, i.e., a silane and siloxane solution (labelled ‘Sol-SIL’) and a mixture of silane and siloxane emulsified in water (labelled ‘Emul-SIL’).

## 2. Results

### 2.1. Stone Characterisation

**Figure 2:**
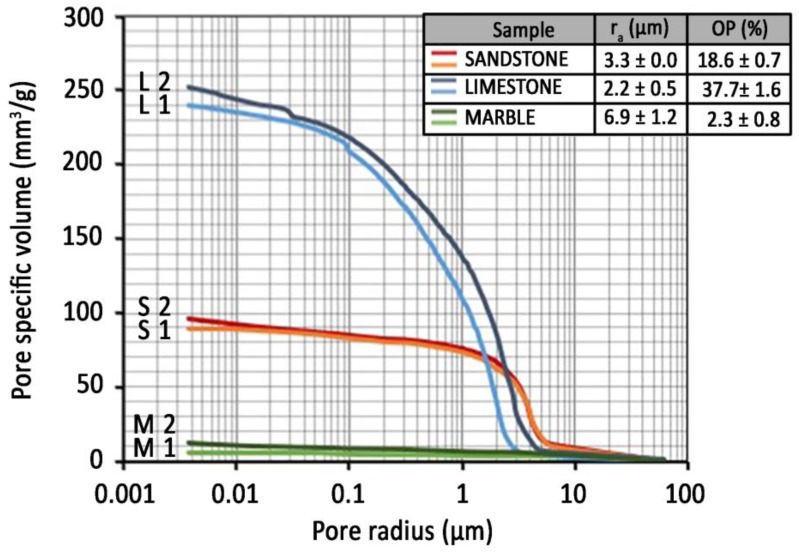
Pore size distribution curves of two samples of sandstone, limestone and marble, obtained by MIP. Open porosity (*OP*) and average pore radius (*r*_a_) in the table were averaged for two samples (L1 and L2 indicate the two limestone samples, S1 and S2 the two sandstone samples, M1 and M2 the two marble samples).

Porosity was determined in untreated and coated stones because it plays a major role in all the degradation phenomena that are related to water absorption and also in the effectiveness of protective treatments. The results of mercury intrusion porosimetry (MIP) analysis of the stones are reported in Figure 2. Sandstone, limestone and marble exhibit very different microstructures in terms of total open porosity (*OP*), mean pore radius (*r*_a_) and pore size distribution. Limestone is characterised by the highest porosity (*OP* = 37.7%), the broadest pore size distribution (significant amount of pores can be noticed between 0.1 μm and 4 μm) and an average pore radius equal to 2.2 μm. Sandstone exhibits a medium–high porosity (*OP* = 18.6%), mean pore radius 3.3 μm and most of the pores are in the radius range 1–5 μm. Finally, marble exhibits the lowest porosity (*OP* = 2.3%) and the largest mean pore radius (*r*_a_ = 6.9 μm).

The high porosity of sandstone and limestone, together with their pore size mostly in the range 0.1–10 μm, makes them vulnerable to salt and ice deterioration [36], both of them made possible by the presence of moisture in the stone, hence the need for protecting these stones by water-repellents arises, in order to avoid the stone powdering, crumbling and flaking that cause a significant loss of heritage material. 

The results of XRD (X-ray Diffraction) analysis and the calcite and dolomite percentages, determined by the Dietrich–Frühling method, are reported in Table 1. 

The three stones are mainly composed of calcite: 88% in sandstone, 86% in limestone and 98% in marble, with the latter also containing 2% dolomite. Furthermore, sandstone contains quartz, while in limestone traces of fluoroapatite were detected. The mostly calcitic composition of these stones makes them susceptible to chemical attack in polluted atmospheres, with consequent formation of black crusts at the expense of the original materials—hence, again, the opportunity for protecting these stones from water (whose presence boosts chemical attack) arises.

### 2.2. Hydrophobicity of the Coated Stones

#### 2.2.1. Water Absorption by Capillarity

The ability of a treatment to reduce the capillary water absorption of stone represents the main goal of any protective; hence, this test can be considered one of the most significant parameters for predicting the real on-site performance of the treatment. The results are presented in the following using the labelling code reported in Table 2. 

**Figure 3:**
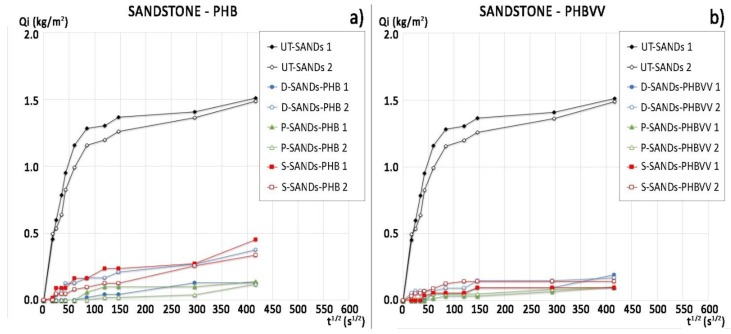
Water absorption curves of sandstone samples treated with: (**a**) PHB-based formulation by dip coating, poultice and spray and of untreated samples (duplicate samples for each condition); (**b**) PHBVV-based formulation by dip coating, poultice and spray and of untreated samples (duplicate samples for each condition).

The water absorption curves of sandstone treated with PHB and PHBVV are reported in Figure 3, together with the curves of the untreated samples (label “UT-“). The slope of the first part of the curve, which is approximately linear, represents the so-called ‘sorptivity’ (capillary absorption rate), while the horizontal part indicates that sample saturation has occurred. The time for the calculation of the ratio of protection (*Rp*) was set at 1 h and at 48 h, as explained in Section 4.5.1, and the results are reported in Table 3. In fact, after 1 h the slope of the first part of the curves notably decreases even if the untreated samples of sandstone do not reach a plateau, but continue to absorb water even after 24 h. The fast absorption of water by sandstone is consistent with its pore size distribution shown in Figure 2. PHB shows a good protective performance, as its *Rp* moves from 87% to 100% after 1 h in contact with water and from 68% to 91% after 48 h. PHBVV shows an even better performance, strongly reducing the absorption of water during all the 48 h (*Rp* ~90%). In both cases, the poultice application gives the best results, probably due to the higher quantity of biopolymer retained on the stone with this method. 

**Figure 4:**
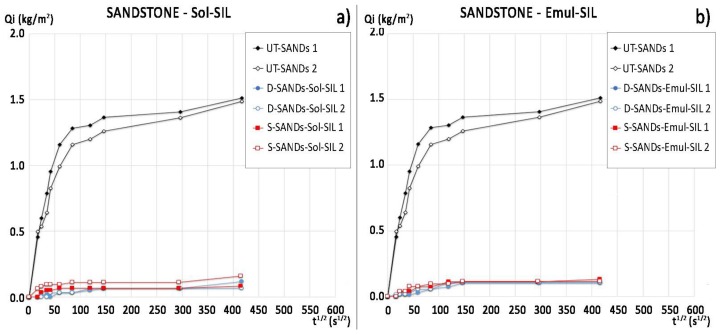
Water absorption curves of sandstone samples treated with: (**a**) Sol-SIL by dip coating and spray and of untreated samples (replicate samples for each condition); (**b**) Emul-SIL by dip coating and spray and of untreated samples (replicate samples for each condition).

The water absorption curves and ratio of protection values (*Rp*) of the commercial protectives are reported in Figure 4 and Table 3, respectively. There are no significant differences between the performance of Sol-SIL and Emul-SIL, as they contain similar polymeric compounds. Both products show high protection (*Rp* varying from 98% to 90% after 1 h and from 89% to 97% after 48 h). The application of the products by dip coating seems to increase their efficacy; in particular, Emul-SIL applied by dip coating can reach a 97% protection ratio after 48 h of testing. The performance of the PHBVV solution, regardless the application method, and the PHB solution applied by poultice, is comparable to that obtained by the two commercial products.

**Figure 5:**
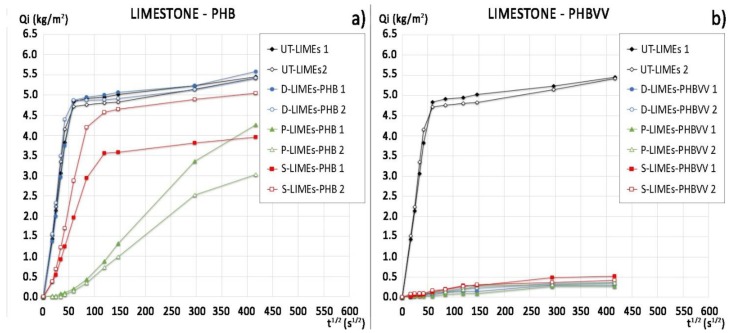
Water absorption curves of limestone samples treated with: (**a**) PHB-based formulation by dip coating, poultice and spray and of untreated samples (duplicate samples for each condition); (**b**) PHBVV-based formulation by dip coating, poultice and spray and of untreated samples (duplicate samples for each condition).

Figure 5 shows the water absorption curves of limestone samples, while the mean ratio of protection values, calculated at 30 min (where the slope of the untreated curves suddenly changes) and at 48 h for all the samples, are reported in Table 4. Limestone shows a relatively high water absorption (final water uptake ~305 kg/m^3^, Figure 5), compared to sandstone (final water uptake ~93 kg/m^3^, Figure 3), in consequence of the significantly higher open porosity. 

**Figure 6:**
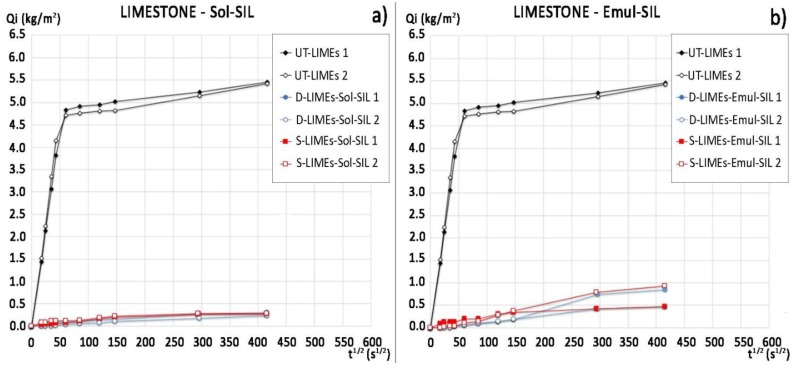
Water absorption curves of limestone samples treated with: (**a**) Sol-SIL by dip coating and spray and of untreated samples (replicate samples for each condition); (**b**) Emul-SIL by dip coating and spray and of untreated samples (replicate samples for each condition).

In this case, the performance of PHB-based protectives is significantly different from that of PHBVV-based. While PHBVV shows excellent capacity to reduce the water absorption regardless the application method used, PHB does not provide significant protection (*Rp* = 0% for PHB applied by dip coating) or protects only in the short term: *Rp* is equal to 98% and 68% after 30 min for PHB applied by poultice or spraying, respectively, but *Rp* is equal to only 43% and 28% after 48 h. The higher effectiveness of the spray application with respect to the dip coating seems due to its more abundant deposition on the surface rather than deeper penetration into the sample. The performance of the two commercial protective products in limestone seems independent from the application method used, as shown by the water absorption curves in Figure 6 and the *Rp* values in Table 4. The protection provided by Sol-SIL is higher than the one given by Emul-SIL, as Emul-SIL strongly reduces the sorptivity in the first 6 h but then its efficacy decreases, while Sol-SIL provides the same protective performance until the end of the test (*Rp* equal to 95% after 48 h). The performance of PHBVV solution applied by dip coating and poultice is comparable to that provided by Sol-SIL.

Capillary absorption test was not performed on marble samples, as their extremely low porosity causes insignificant water absorption, even for the untreated samples.

#### 2.2.2. Contact Angle Measurements

The hydrophobicity induced on the stone surface was evaluated by means of both static and dynamic contact angle measurement, to obtain a reliable evaluation of samples water wettability. The contact angles of PHB and PHBVV alone had been previously determined on glass slides immersed in the polymer solution and let to evaporate (solvent casting), obtaining the following values:

- PHB: static contact angle 88° ± 1°; dynamic contact angle (advancing) 90° ± 1°; dynamic contact angle (receding) 56° ± 3°;
- PHBVV: static contact angle 88° ± 1°; dynamic contact angle (advancing) 92° ± 1°; dynamic contact angle (receding) 63° ± 2°.

Table 5, Table 6 and Table 7 report the contact angle values for sandstone, limestone and marble, respectively. Static contact angle values measured on the untreated samples show huge differences in the three substrates: marble exhibits the highest contact angle (*θ* = 41° ± 7°), followed by sandstone (*θ* = 15° ± 4°) and limestone, for which an immediate and complete absorption of the drop occurs (*θ* = 0° ± 0°). These differences are related to both the chemical composition of the three stones and their surface roughness and porosity. It is actually not straightforward to characterise non-ideal solid surfaces (i.e., chemically heterogeneous and porous) through static contact angle measurements, because on such surfaces the only measurable value is the apparent contact angle, which can be largely different from the ideal contact angle [2,9,37,38]. However, for the purposes of this study, the effects of porosity and chemical non-homogeneity on the contact angle were not addressed in detail. 

Sandstone treated with PHB exhibits static contact angles slightly above 90°, which is considered the borderline value between a hydrophobic (*θ* > 90°) and a hydrophilic behaviour (*θ* < 90°), hence its performance is satisfactory even if not outstanding. Conversely, PHBVV-based protective shows a static contact angle between 90° and 125° (Table 5), hence markedly hydrophobic behaviour. The best improvement was given by PHBVV applied by poultice, but in all the other samples treated by PHB and PHBVV the application method was not found to play a key role. Sol-SIL induces the highest hydrophobicity (*θ* = 140°), while the performance of Emul-SIL is comparable to that of PHBVV. Standard deviation values are higher for PHA formulations than for the two commercial products, suggesting that the latter more homogeneously distribute on the stone’s surface.

For limestone (Table 6) the PHAs formulations produce the highest improvement of static contact angle with respect to sandstone and marble, starting from the condition of complete absorption of the untreated samples (*θ* = 0°) and reaching values between 110° and 125°. As for sandstone, Sol-SIL gives the highest values of contact angle (*θ* > 140°), while the performance of Emul-SIL is comparable to that of PHBVV. Again, standard deviations for PHA formulations exceed those of the two commercial products.

The static contact angles measured on marble samples treated by PHB and PHBVV are doubled with respect to the untreated stone (Table 7), but they do not reach 90°, hence not showing proper water-repellent behaviour. The only exception is PHBVV applied by poultice (*θ* = 109° ± 10°). Instead, both Sol-SIL and Emul-SIL make static contact angle reach values around 120°, although their standard deviation is here comparable to those of untreated stone and of stones treated with PHB and PHBVV. This quite high standard deviation can be due to a lower homogeneous coverage of the marble surfaces by means of commercial protective treatments with respect to sandstone and especially limestone, possibly correlated to the low roughness of the marble, which notably reduces the presence of anchorage points useful for coating adhesion and to the full calcitic composition of marble, which does not promote the chemical bonding with the silicon-based protectives.

Table 5, Table 6 and Table 7 also report the values of advancing contact angles, determined by dynamic measurement. As expected for rough and non-homogenous surfaces, for the untreated stones the values of static and advancing contact angles are quite different. Conversely, the advancing and static contact angles are in fairly good agreement in all samples treated with PHB- and PHBVV-based formulations, being the advancing contact angle very close to the static one or slightly higher (difference less than 10°). This is representative of the capability of the PHAs-based formulation to enter into the surface porosity and modify the surface chemistry of the stones. The same consideration can be done for sandstone and marble samples treated by Sol-SIL and Emul-SIL, while limestone exhibited very high advancing contact angles, in the range between 140° and 166°, and generally higher than the respective static contact angle.

Results clearly show that all the protectives applied on marble lead to poor improvements, due the very low porosity of the starting substrate. 

As regards to receding contact angles measured for sandstone and limestone, only few samples treated with PHB or PHBVV exhibited receding contact angles higher than 0°, but significantly lower than 90° (being equal or lower than 26°, Table 5 and Table 6). The commercial product Sol-SIL reached receding contact angles included between 25° and 48°, higher than those given by the PHAs formulations (Table 5 and Table 6). The commercial product Emul-SIL gives similar results of Sol-SIL if applied to sandstone, while 0° of receding contact angle if applied to limestone (Table 5 and Table 6). 

Marble is the only stone that recorded, as untreated stone, a receding contact angle higher than 0° (being equal to 19°, Table 7). Due to that, samples treated with PHB-, PHBVV-formulation and commercial products exhibited contact angles higher than 0° and included between 8° and 40°. However, a significant improvement of the receding contact angle for the treated stones is not evident, with the receding contact angle being in some cases lower than the one obtained for the untreated sample. 

Advancing contact angle represents the upper limit of every possible contact angle configuration; hence, it is expected to be influenced by the presence of any protective treatment. For this reason, a high increase of advancing contact angle with respect to the untreated samples confirms the presence and action of the protective on the stone’s surface [2,9,37]. Instead, receding contact angle is considerably influenced by the presence of defects and heterogeneity, which are correlated both to the stone mineralogical composition and to incomplete coverage of the stone substrate by the polymer [2,9,37]. However, the roughness of stone inevitably causes a certain amount of hysteresis between advancing and receding contact angles. Hence, although a good protective should theoretically provide the stone with high dynamic contact angles (both advancing and receding angles >90°), a certain amount of water may be absorbed by the stone by capillarity (possible, despite the treatment application) or may be retained in the stone roughness during the measurements of dynamic contact angles. For this reason, the argument of the arccosine function *F*_0_/*L*γ in Equation (3) (Section 4.5.2) may happen to exceed 1, as the presence of water increases the sample mass and so the value of *F*_0_. In this case, the contact angle calculation leads to a value equal to 0° even if, from a trigonometric point of view, the equation cannot be solved. This tricky aspect of contact angle calculation is due to the fact that the Wilhelmy theory used for contact angle measurements with the force tensiometer does not take into account water absorption or entrapment during the test. As a result, the 0° receding contact angles reported in Table 5 and Table 6 actually derive from values of *F*_0_/*L*γ > 1 and it should be concluded that these stone samples, due to their porosity, heterogeneity and roughness, are not suitable for receding contact angle measurement by means of force tensiometer. 

The comparison between results obtained by static and dynamic contact angle measurement and by capillary water absorption test can be useful to clarify the entire performance of protective treatments applied on stone substrates. 

Sandstone samples treated by PHB generally exhibit a relatively good performance in terms of dynamic contact angle (advancing angle > 100°, Table 5), water absorption (with the lowest *Rp* equal to 75% after 48 h, Table 3) and static contact angle (slightly higher than 90°, Table 5). PHB applied by dip coating gave very good results in terms of water absorption, with *Rp* equal to 94% after 1 h and 84% after 48 h (Table 3). However, PHBVV gave even better results in terms of capillary water absorption (Figure 3b) and advancing and static contact angles when applied by dip coating and poultice (both angles > 100°, Table 5). Static and dynamic contact angles after treatment by Sol-SIL and Emul-SIL are comparable and maximum for this type of substrate (*θ* equal to 140° for Sol-SIL and 125° for Emul-SIL, *θ*_adv_ between 135°and 145°, Table 5). The same consideration can be made on their performance in terms of water absorption by capillarity (with *Rp* comprised between 89% and 97%, Table 3).

For limestone, the performance of PHB is good in terms of static and advancing contact angles (comprised between 110° and 125°, Table 6), but not fully satisfactory in terms of capillary water absorption (*Rp* < 40%, Table 4). In the case of PHBVV, there is a good agreement between the performance evaluated in terms of capillary water absorption and wettability: the great reduction in water absorption (*Rp* > 90%, Table 4) is accompanied by high contact angles (with the static and advancing ones between 120°–125°, Table 6). Sol-SIL gave the best results both in terms of wettability (static and advancing contact angles > 140°, Table 6) and reduction of capillary water absorption (*Rp* equal to 95% after 48 h, Table 4). Also Emul-SIL gave very good results on this stone (Table 4 and Table 6).

Thus, in light of the present results, a good performance in terms of wettability does not always correspond to a good performance in terms of capillary water absorption and vice versa. Moreover, low (*θ*_rec_ < 25°) or zero receding contact angles are generally not correlated with high water absorption, as explained above. This highlights the importance of analysing different aspects concerning protective performances. 

### 2.3. Colour Measurement

**Figure 7:**
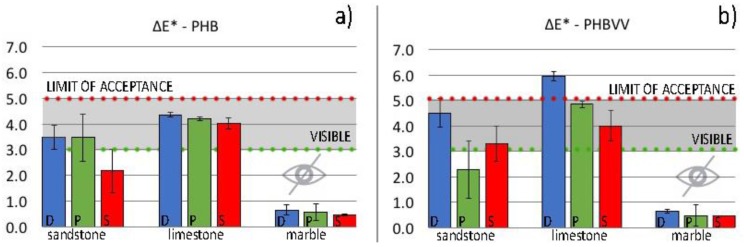
Colour alterations (ΔE*) determined by spectrophotometry on sandstone, limestone and marble: (**a**) before and after the application of PHB-based treatment; (**b**) and before and after the application of PHBVV-based treatment. The three application methods are represented as D (dip- coating, blue bars), P (poultice, green bars) and S (spray, red bars).

Colour alteration values, determined by spectrophotometer and calculated on the basis of the coordinates in the CIELAB space (ΔE*), are reported in Figure 7 for stone treated with PHB and PHBVV, with respect to untreated ones. The CIELAB colour space was established by the “Commission Internationale de L’Eclairage” (CIE) in 1976 and allows to represent each colour by the three coordinates L* (axis black-white), a* (axis green-red) and b* (axis yellow-blue). The difference between two colours can be determined by the formula ΔE* = (ΔL*^2^ + Δa*^2^ + Δb*^2^)^1/2^.

For colour compatibility in the conservation field, any consolidating or protective treatment is required to produce a ΔE* lower than 5, considering that the human eye cannot detect colour alterations with ΔE* < 2–3. 

Results show that PHB formulation (Figure 7a), regardless the application method, gives acceptable values of ΔE* both for sandstone (values between 2 and 3.5) and limestone (values about 4), while colour variations for marble are imperceptible to the human eye (ΔE* lower than 1). After the application of PHBVV, the colour alteration is acceptable for sandstone samples (ΔE* between 2 and 4.5) and marble (undetectable by human eye) (Figure 7b), while for limestone, ΔE* is higher than the threshold for dip coating application (ΔE* ≈ 6). 

Based on these results, PHB- and PHBVV-based treatments can be considered compatible from an aesthetic point of view with all the stones considered. Dip coating application seems to give systematically higher colour changes with respect to the other methods, but only in one case (PHBVV) did it produce excessive colour alteration. This higher colour impact of dip coating cannot be ascribed to a higher amount of protective on the surface, because this is not the case; hence, a deeper investigation into the surface distribution of the polymer will be necessary to find an explanation of this aspect. 

In Table 8 the average variations ΔL*, Δa* and Δb* are reported, for a better understanding of colour alterations. The values that mostly influence ΔE* are Δb*, indicating a yellowing of the surface, and ΔL*, indicating a darkening. 

### 2.4. Water Vapour Diffusion Test

Results of the water vapour diffusion test for stone treated with PHB and PHBVV are reported in Table 9, Table 10 and Table 11, where the water vapour transmission rate of the untreated substrate (*V*_S_), of the treated substrate (substrate plus coating, *V*_CS_), of the coating (*V*), the water vapour diffusion-equivalent air layer thickness (*S*_d_) and the corresponding water vapour transmission rate class are collected. In order to obtain good compatibility between the protective treatment and the stone substrate, it is essential not to significantly alter the water vapour diffusion of the stone. Due to the different microstructure, untreated sandstone, limestone and marble have notably different water vapour diffusion rates (*V*). In particular, limestone exhibits the highest V (278 g/m^2^·day, Table 10), which is more than three times that of sandstone (86 g/m^2^·day, Table 9) and more than 13 times higher than that of marble (21 g/m^2^·day, Table 11).

In sandstone, the results of water vapour diffusion testing show good compatibility between the coatings and the stone, as every coating applied on sandstone can be classified in the high water vapour diffusion rate class, except for PHB and PHBVV applied by poultice. These give a medium water vapour diffusion rate class, probably related to the high quantity of polymer retained in the stone after poultice (Table 9). Nevertheless, even in the case of poultice application of PHB and PHBVV, the water vapour transmission rate of the stone is reduced by less than 30% (being *V*_S_ = 86 g/m^2^·day and *V*_CS_ = 59–60 g/m^2^·day for samples treated with PHB and PHBVV by poultice, Table 9). 

PHB and PHBVV treatments applied on limestone are classified in the high water vapour diffusion rate class (Table 10). Nevertheless, two samples, namely S-LIMEs-PHB and D-LIMEs-PHBVV, gave values of water vapour transmission rate (*V*_CS_) that are notably lower with respect to the other samples (being, respectively, *V*_CS_ = 163 g/m^2^·day and *V*_CS_ = 126 g/m^2^·day versus *V*_CS_ > 260 g/m^2^·day for the other samples). This cannot be due to a higher amount of protective applied, but seems related to the considerable heterogeneity that characterises stone samples and, in particular, limestone [39]. 

All the coatings applied on marble belong to the medium class of water vapour diffusion (Table 11), but given the extremely low water vapour diffusivity of marble, the significance of this parameter is quite limited and even a medium water vapour transmission rate can be considered compatible. For the same reason, the fact that the values of water vapour transmission rate after PHBVV application are comparable or even higher with respect to the untreated stone appears simply due to the difficulty of determining accurately the water vapour diffusion in this very compact stone. 

Based on the results, the compatibility from the point of view of water vapour transmission capacity is ensured for both the PHB and PHBVV formulations and for all the stones investigated. 

### 2.5. Coating Morphology Analysis

Treated samples showing the best performance in terms of water repellency were analysed by scanning electron microscopy in order to evaluate the interfacial adhesion between the protective coating and the stone, and the coating homogeneity. Of course, the poultice method gives rise to the thickest coatings because it allows us to deposit a greater amount of protective solution onto the surface; therefore, samples produced by the poultice method were used for the purposes of this specific analysis.

**Figure 8:**
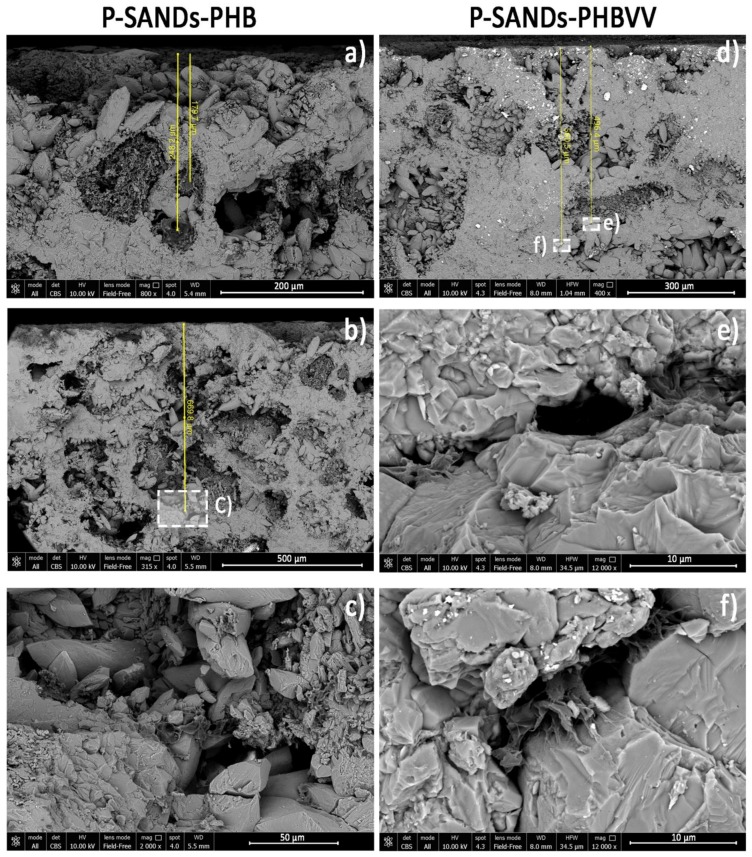
FEG-SEM images of sandstone treated: (**a**–**c**) with PHB-based formulation by poultice; (**d**–**f**) with PHBVV-based formulation by poultice.

**Figure 9:**
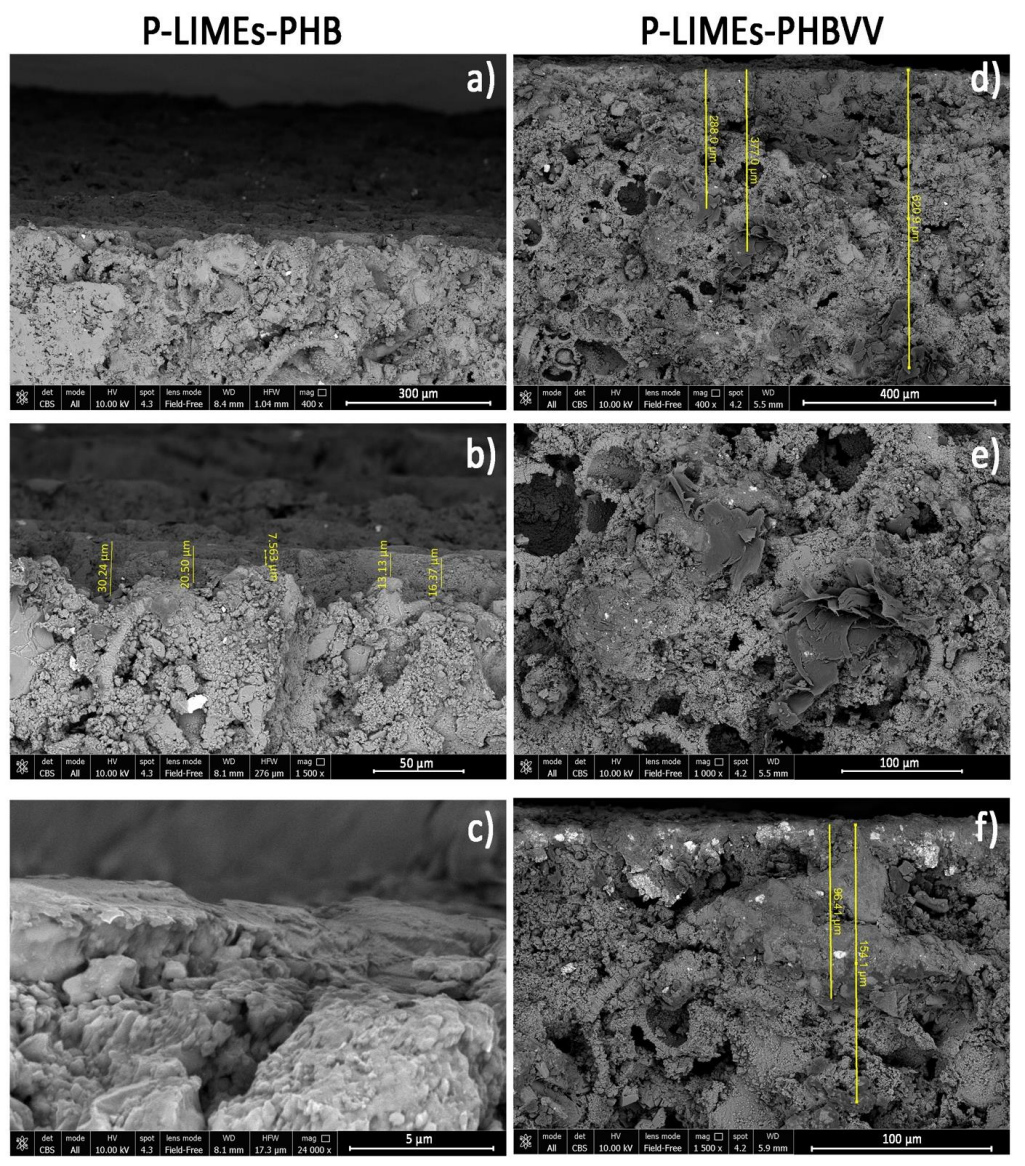
FEG-SEM images of limestone treated: (**a**–**c**) with PHB-based formulation by poultice; (**d**–**f**) with PHBVV-based formulation by poultice.

**Figure 10:**
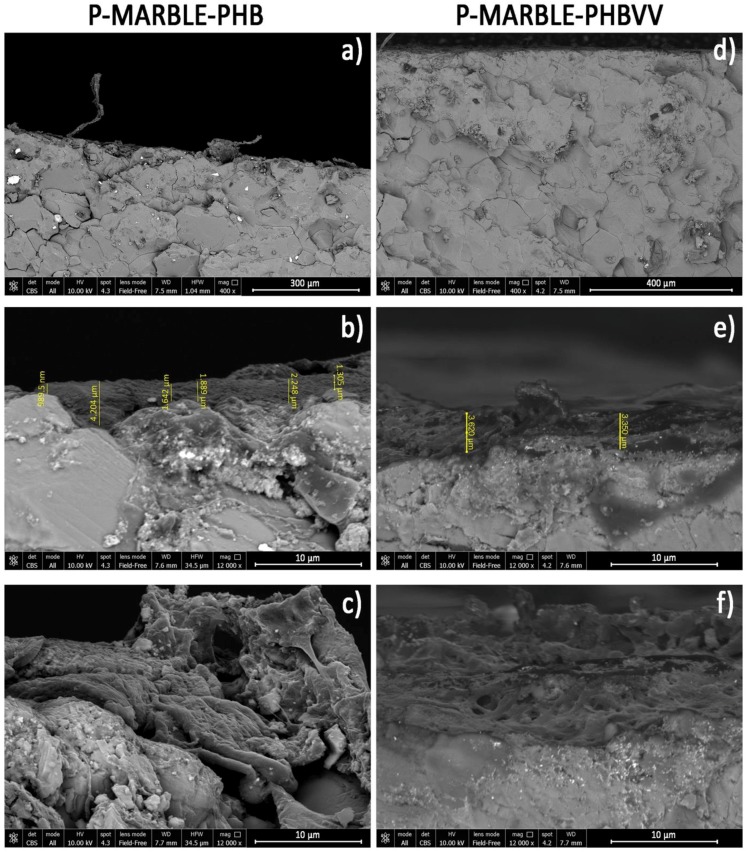
FEG-SEM images of marble treated: (**a**–**c**) with PHB-based formulation by poultice; (**d**–**f**) with PHBVV-based formulation by poultice.

Some images obtained by FEG-SEM (Field Emission Gun Scanning Electron Microscopy) for sandstone, limestone and marble samples treated with PHB and PHBVV by poultice are reported in Figure 8, Figure 9 and Figure 10. In sandstone, no significant morphological differences between the PHB and PHBVV coatings can be noticed. In both cases the polymer tends to penetrate the capillary pores of the stone, assuming a shape similar to a cobweb. Pores are not totally filled by the polymer, suggesting that the treatment does not give a pore blocking effect. The polymer is present at all the observed depths (approximately 650 μm) and as a thin layer over the top of the surface (dark layer in Figure 8b). Images of treated cross sections of limestone (Figure 9) suggest a distribution of the polymer similar to that observed in sandstone, but in this case the morphology of the polymer in the pores is sheet-like. Due to the very low porosity of marble, both PHB and PHBVV accumulate in layers of various thickness (1–4 μm) over the stone’s surface (Figure 10).

### 2.6. Artificial Ageing

**Figure 11:**
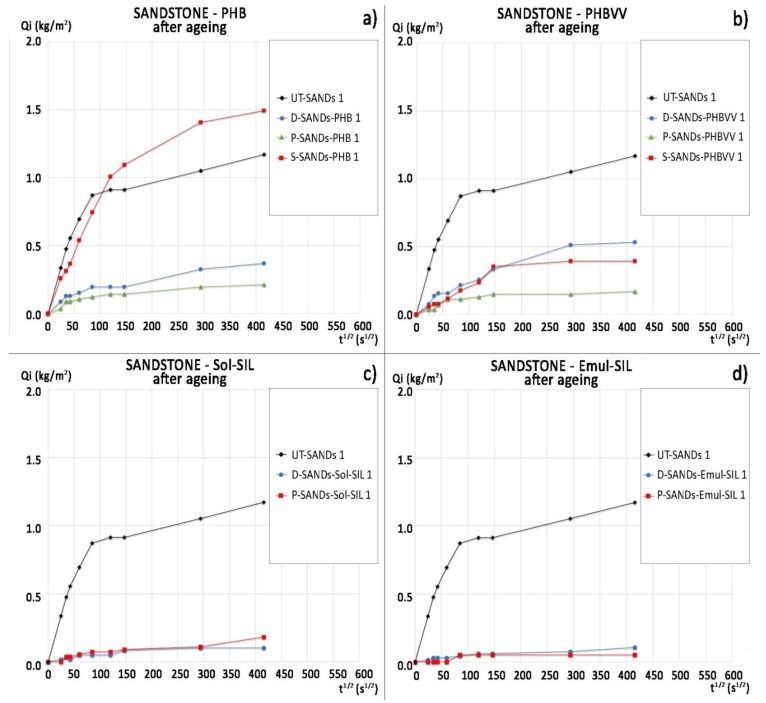
Capillary water absorption curves of sandstone after artificial ageing referred to: (**a**) samples treated with PHB-based formulation; (**b**) samples treated with PHBVV-based formulation; (**c**) samples treated with Sol-SIL; (**d**) samples treated with Emul-SIL.

**Figure 12:**
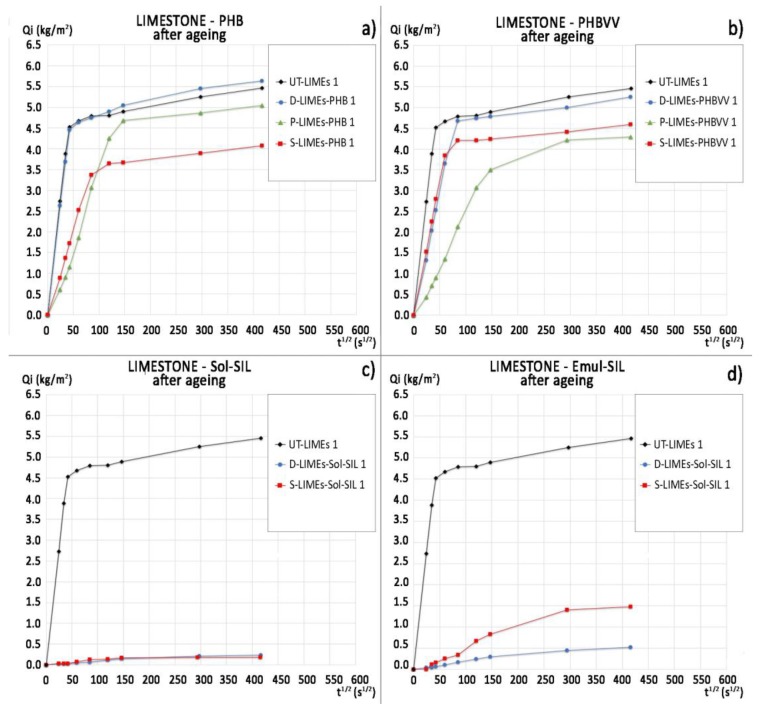
Capillary water absorption curves of limestone after artificial ageing referred to: (**a**) samples treated with PHB-based formulation; (**b**) samples treated with PHBVV-based formulation; (**c**) samples treated with Sol-SIL; (**d**) samples treated with Emul-SIL.

The results of static contact angle measurement after seven days of artificial ageing in the climatic chamber are reported in Table 12, where a drastic decrease of water repellency can be observed for PHB and PHBVV, while the commercial products experience a limited decrease in the contact angle (especially Sol-SIL). However, considering the water absorption by capillarity, PHB and PHBVV seem to provide some residual effectiveness on sandstone even after the artificial ageing (Figure 11 and Table 13), while for limestone the loss of hydrophobicity is confirmed (Figure 12 and Table 14).

These preliminary results suggest that measures must be taken for improving the durability of these PHB and PHVV formulations for the application targeted in this study (for example, adding additives and stabilisers).

It is noteworthy that one of the most important features of PHAs is their spontaneous degradation under environmental conditions; therefore, the results obtained by the accelerated ageing should not come as any surprise, but on the contrary demonstrate that spontaneously reversible surface treatments for stones can be successfully developed using bioplastics. This actually represents a very important target for the protection of stones in cultural heritage, where tailoring of the duration of the treatment can be addressed by a proper selection of the molecular features of the biopolymer chains, because they directly influence the environmental duration of the coating.

## 3. Discussion

A preliminary investigation into the possible use of PHAs for the protection of stone in cultural heritage was carried out. Results demonstrated that PHAs can be purposely used as a polymer basis for the development of protective coatings for different kinds of stone (sandstone, limestone and marble were tested), and their intrinsic biodegradability in environmental conditions can be purposely exploited to generate temporary treatments that do not need any removal, which is an important target for the protection of cultural heritage. Results showed that the molecular structure of the PHAs does not play a fundamental role, even if PHBVV usually gives rise to slightly better results than PHB. 

Experiments demonstrate that the application method, together with the porosity of the stone, strongly influences the amount of polymer deposited on the stone, and the effectiveness of the protective treatment as a consequence. Limestone, given its higher porosity, retains a higher amount of protective treatment than sandstone; therefore, more significant improvement in hydrophobization is reached. As far as the deposition method is concerned, poultice causes a much higher protective uptake with respect to dip coating and spray, in porous stones as in sandstone and limestone, with the uptake being maximum for the latter. In the case of marble, given its extremely low porosity, a very limited uptake was observed for any protective and any application method, so improvements induced by the presence of the protective are limited. 

The PHAs-based protective formulations generally give good results in terms of colour change and water vapour diffusion. Only in one case (PHBVV applied by dip coating to limestone) was the colour change slightly higher than the threshold accepted in the conservation field. The water vapour transmission rate class was generally ‘high’ in sandstone and limestone. 

Investigating the performance of protectives on real stone samples is very challenging, as each of the testing methods used provides only partial insight into the expected performance on site. For this reason, it is very important to develop a testing procedure that is actually able to reproduce in the lab the protective performance that is expected in real on-site exposure. In particular, the use of a force tensiometer might be too severe in relation to the real condition of stone on-site, which does not involve a complete immersion in water. From this point of view, the capillary absorption test can be considered more representative, although the water in the test is supplied by interposition of a wet layer of filter papers rather than by plain water or rainfall. For these reasons, it would be useful to develop new test methods targeted to investigate in a more realistic way the performance of protective treatments for stone, for example by simulated artificial rain tests.

Further optimisations of the biopolymer-based formulations, mainly looking for ‘greener’ solvents in substitution of chloroform and adding stabilisers for tailoring the polymer durability, are currently in progress.

## 4. Materials and Methods

### 4.1. Stone Samples

The three lithotypes used for the purposes of this study were:

- Sandstone: a medium-porosity calcitic sandstone, Siena stone, was selected (provided by Il Casone S.p.A., Firenzuola, Italy). It is mainly composed of calcareous grains and low amounts of quartz, bound by calcitic cement. This stone is typical of Tuscany architecture, but representative of a class of stones widely used in historical architecture.
- Limestone: a high porosity organogenic calcareous stone, Lecce stone, quarried in the Lecce area in Italy (Cursi-Zollino-Melpignano quarry) and provided by Décor s.r.l., San Giovanni in Fiore, Italy), was selected. It is mainly composed of calcite, with traces of phosphatic minerals. This limestone was widely used in Baroque architecture in the Puglia region and is similar to several other porous limestones widespread in the Mediterranean basin.
- Marble: Carrara marble, a very low porosity stone (supplied by Imbellone Michelangelo, s.a.s. Bologna, Italy), quarried in the Apuan Alps in Tuscany and widely used in historical architecture and statues, was selected. It is mainly composed of calcite, with small traces of dolomite.

Stone samples were obtained by wet sawing of quarried slabs. Sample size and geometry were different according to the type of test to be performed. Before the application of any protective treatments, the samples were gently brushed under water, kept in an oven at 40° C for 24 h and then in laboratory conditions until constant weight. 

### 4.2. Stone Characterisation

The microstructure of the substrates was investigated in terms of pore size distribution, total open porosity (*OP*) and average pore radius (*r*_a_), by mercury intrusion porosimetry (MIP) on duplicate fragments (about 1 g mass) per stone type. For this purpose, a Fisons Macropore Unit 120 and a Porosimeter 2000 Carlo Erba (Tecmat, Como, Italy) were used. 

The mineralogical composition of each stone was determined by powder X-ray diffraction (XRD), in a Philips Diffractometer PW 1840 (Panalytical, Almelo, The Netherlands), 40 kV/20 mA, Cu Kα radiation. The carbonate amount, expressed as CaCO_3_ (wt %), was determined on duplicate samples by the Dietrich–Frühling gas volumetric method. This method is based on the quantification of the CO_2_ volume released by reacting the powdered sample with HCl. The method also allows for dolomite quantification [40], as the reaction velocity between HCl and dolomite is lower than that between HCl and calcite, so it is possible to distinguish between them. 

For any stone type and condition, all the tests (described here and in Section 4.5) were carried out on two duplicate samples. 

### 4.3. Protective Formulations

PHB and PHBVV were kindly provided as experimental grades by Bio-on SpA (Bologna, Italy) with an average ponderal molecular weight of Mw = 122,500 and Mw = 279,500, respectively; the ponderal molecular weight was determined by gel permeation chromatography (GPC) analysis dissolving 10 mg of sample (powder) in 2 mL of chloroform and using toluene as a flow marker (Chromatographyc system Agilent 1260 Infinity System by Agilent, Santa Clara, CA, USA, based on two columns: PLgel MiniMIX-A for the separation of molecules with molecular weight up to 4 × 10^7^ g/mol and TOSOH TSKgel SuperMultipore HZ-M for the separation of molecules with molecular weight in the range 10^2^–2 × 10^6^ g/mol); both were used as received without any further purification. The molar content of 3-hydroxyvaleric acid (3HV) and 4-hydroxyvaleric acid (4HV) units of PHBVV was determined by means of Bruker NMR Avance400 spectrometer (Bruker, Billerica, MA, USA) and the software Bruker TopSpi (version 3.2, Bruker, Billerica, MA, USA), using 10–15 mg of sample (powder) dissolved in 1 mL of CDCl_3_. PHBVV has a 3HV units molar content of 11% and a 4-HV units molar content of 2%. The melting temperature of the PHB used for this study was 173 °C, and 146 °C for PHBVV, hence both polymers can be considered suitable for exterior applications; glass transition temperature was not detectable by DSC for PHB due to its high crystallinity, while it was −2 °C for PHBVV. Homogeneous solutions of PHAs were obtained by dissolving the polymers in boiling CHCl_3_ at a concentration of 3 wt/vol %; cold solutions were strained with a syringe filter (0.45 μm) before use, in order to eliminate any possible insoluble traces. At this preliminary stage of the research, aimed at evaluating the potential of PHB and PHBVV as protectives, chloroform was used as the solvent (despite not being usable in the workplace), as it is known to be effective for this class of biopolymers, exhibiting a low solubility in many classical polymer solvents.

Two commercial protectives widely used for stone conservation were tested too, for comparison’s sake. The first one, labelled Sol-SIL, is constituted by silane and siloxane dissolved in white spirit (active content of 7 m/m%, commercial name Idrosil^®^ Pronto CA-WS, by Antares, San Lazzaro di Savena, Italy); the second, labelled Emul-SIL, is constituted by silane and siloxane emulsified in water (active content of 8 m/m%, commercial name Antipluviol^®^ W, Mapei, Milano, Italy).

### 4.4. Application Methods

The PHB- and PHBVV-based treatments were applied by:

- Dip coating (sample coding: “D-”): samples were completely immersed in the solution for 10 min. The samples destined to capillary water absorption test and water vapour permeability determination were only partially immersed in the solution for 10 min (keeping them suspended from the top), in order to obtain just one treated surface.
- Poultice (sample coding: “P-”): the sample surface to be treated was covered with a 1.5-thick layer of cotton wool, then the formulation was spilled over the cotton layer (0.16 L of solution per 1 dm^2^ of treated surface) and the samples were immediately covered with an aluminium sheet to prevent the solvent from evaporating. The poultice was left wrapped for 24 h, to allow the absorption of the protective in the stone. Thereafter, the aluminium sheet was removed and the cotton layer was left over the samples until complete drying. Only one surface was treated per each sample, except for samples prepared for the measurement of static and dynamic contact angle, which were completely covered by a poultice.
- Spray (sample coding: “S-”). A low-pressure spray nebuliser (FPM gaskets industrial sprayer, Volpitech 2, Volpi, Casalromano, Italy) was used for this purpose. The surfaces of the samples to be treated were put in vertical position and subjected to 15 sprays, corresponding to about 0.02 L of solution per 1 dm^2^ of surface. The distance between the nozzle and the sample was about 40 cm. Only one surface per each sample was treated, except for samples prepared for the measurement of static and dynamic contact angle, which were completely sprayed.
- Sol-SIL and Emul-SIL were applied by spray, as recommended by the manufacturers, but also by dip coating, for comparison’s sake, using the procedures previously described.

### 4.5. Characterisation of the Coated Stones

#### 4.5.1. Capillary Water Absorption

The water absorption by capillarity was determined according to EN 15801 [41] on two replicate samples (25 × 25 × 19 mm^3^) for each combination of formulation (PHB-based or PHBVV-based) and application method (dip coating, poultice and spray). Two untreated samples were tested for reference. Samples were put in contact with a 1 cm-thick layer of filter paper immersed in deionised water up to the half of its thickness, then weighed at fixed intervals of time, until 48 h. The ratio of protection by capillarity (*Rp* %) was calculated as:

(1) $$Rp\;\%=\frac{Q_{\mathrm{UT}}-Q_{T}}{Q_{\mathrm{UT}}}\times 100,$$

where *Q*_UT_ and *Q*_T_ are, respectively, the mean mass of water absorbed by the untreated and the treated sample at the time when, according to [42], the plateau of absorption is reached. The ratio of protection by capillarity (*Rp* %) was also calculated referring to 48 h of test. As *Rp* is calculated from the mean mass of water absorbed by the untreated and treated samples (two duplicate samples for each condition), the standard deviation is not reported for this parameter in Table 3, Table 4, Table 13 and Table 14. 

#### 4.5.2. Contact Angle Measurement

Samples used for the static and dynamic contact angle measurements were slabs sized 30 × 25 × 2.5 mm^3^, in which all the faces were treated. To eliminate powder or non-adherent particles from the surfaces, samples were gently sprayed with clean compressed air before testing.

The static contact angle measurement was performed by using water as the drop phase; the sessile drop method was used, and drop profiles were analysed by means of a OCA system (Dataphysics Contact angle system, software SCA20, Filderstadt, Germany); a drop volume of 3 μL was used. Results are the mean of at least 10 measurements carried out on different points of the stones’ surfaces. 

Dynamic contact angle measurement was performed at room temperature using a force tensiometer Sigma 700 (Biolin Scientific, Västra Frölunda, Sweden) and the results were elaborated by One Attension software (Biolin Scientific) for the calculation of the advancing (*θ*_adv_) and receding (*θ*_rec_) contact angles, considering the steady-state conditions. 

**Figure 13:**
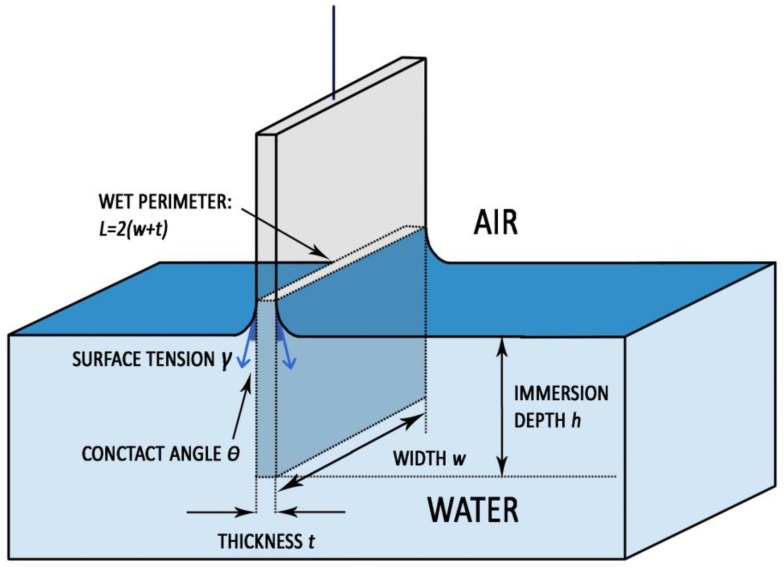
Schematic representation of surface tension acting during the sample immersion and emersion in water by means of force tensiometer when *θ* < 90°. For clarity’s sake, the surface tension effect was represented only for the longer sides of the wet perimeter.

The tensiometer measures the variations of force that occur during the sample immersion and emersion from water. These variations are correlated to buoyancy and to the surface tension, as represented in Figure 13 [37]. The water surface tension acts along the immersed perimeter of the stone samples and it is tilted with respect to the z axis of *θ*_adv_, during immersion, and *θ*_rec_, during emersion. In correspondence of the zero depth of immersion the buoyancy is equal to zero. The instrument returns a graph that has on the x axis the depth of the immersion of the sample in water and, on the y axis, the value of the force (*F*) recorded during the test divided for the wet perimeter of the sample (*L*). Carrying only the linear trend of the force variations to the zero depth of immersion, the extrapolated value of force (*F*) depends only on the surface tension of water (*γ*) acting along the wet perimeter (*L*) and projected in the direction of the force measurement:

(2) $$F_{0}=L\gamma cos\theta .$$

The contact angle is the only unknown parameter and can be calculated by applying the reverse equation:

(3) $$\theta =arccos\frac{F_{0}}{L\gamma }.$$

The immersion and emersion speed was set at 10 mm/min, while the immersion depth was fixed at 10 mm, in order to obtain sufficiently averaged results (and for this reason, no standard deviation values are reported in Table 5, Table 6 and Table 7). Although some researchers recommend a lower speed of immersion/emersion [2,9,37,43], the value of 10 mm/min was selected here to reduce the effect connected to some possible water absorption during the test. 

Differently from the static contact angle measurement, the dynamic one gives actually a range, where the advancing and the receding contact angles represent, respectively, the maximum and minimum values that apparent contact angle may assume, and this provides a more complete understanding of sample wettability. Moreover, the results are averaged along the entire surface immersed in water; hence, they are expected to overcome some of the problems connected to punctual measurement.

#### 4.5.3. Colour Measurement

Colour measurements were performed on slabs (30 × 20 × 2.5 mm^3^), before and after the application of the treatments. For this purpose, a portable spectrophotometer with sphere geometry (model SP62, X-rite, Grand Rapids, MI, USA) with an aperture of 8 mm was used. The colour alteration (ΔE*) produced on each stone by the treatments was determined using the L*, a* and b* coordinates in the CIELAB space [44].

For each sample, two measurements of L*, a* and b* were collected both before and after the treatment, and the mean values of L*, a* and b* were calculated. From these latter values the colour alteration (ΔE*) was calculated. The same procedure was applied to the sample duplicate and the average colour alteration was then calculated. 

#### 4.5.4. Water Vapour Diffusion Test

Water vapour diffusion test was performed using the “wet-cup method” according to ISO 7783 [45]. For each combination of formulation and application method, the test was performed on one prismatic sample (50 × 50 × 20 mm^3^). Given the large area under testing, one sample was considered representative for investigating the water vapour diffusion of the untreated and treated stones. The water vapour transmission rate of each coating, *V* (g/m^2^·day), was calculated as a function of the water vapour transmission rate of the coating plus substrate (*V*_CS_) and of the water vapour transmission rate of the untreated substrate (*V*_S_), following the procedure proposed for non-self-supporting coatings in the cited standard:

(4) $$V=\frac{V_{\mathrm{CS}}\times V_{S}}{V_{S}-V_{\mathrm{CS}}}.$$

Moreover, the water vapour diffusion-equivalent air layer thickness, *S*_d_ (m), was calculated for each sample according to the equation:

(5) $$S_{d}=\frac{\delta_{a}\times \Delta_{PV}}{V},$$

where *δ*_a_ is the water vapour permeation coefficient of air at standard temperature and pressure and ∆_PV_ is the difference between the partial water vapour pressure in the test cup and that in the test enclosure. After determining *V* and *S*_d_, it is possible to classify the transmission rate of water vapour according to EN 1062-1 [46] in:

- high water vapour transmission rate class (*V*1), if *V* > 150 g/m^2^·day and *S*_d_ < 0.14 m;
- medium water vapour transmission rate class (*V*2), if 15 g/m^2^·day < *V* ≤ 150 g/m^2^·day and 0.14 m ≤ *S*_d_ < 1.4 m;
- low water vapour transmission rate class (*V*3) if *V* ≤ 15 g/m^2^·day and *S*_d_ ≥ 1.4 m.

#### 4.5.5. Scanning Electron Microscopy

The morphology of the cross-section of surface treated prismatic stone samples (10 × 15 × 10 mm^3^) was observed by FEG-SEM (FEI Nova NanoSEM 450, Thermo Fisher Scientific, Waltham, MA, USA). Stones treated with PHB- and PHBVV-based formulations applied by poultice on one surface were observed. The cone for back-scattered electrons was set to the widest opening in order to obtain morphological images and, at the same time, the definition of the contrast in function of the chemical composition given by the back-scattered electrons (BSE) mode. The main purpose of the observation of the cross sections was to investigate not only the morphology of the coatings, but also their possible penetration in the porosity of the samples.

#### 4.5.6. Accelerated Ageing

Treated samples were kept for seven days in a climatic chamber (Discovery chamber DY340, by Angelantoni Industrie S.p.A., Cimacolle, Italy, ACS Environmental testing division) at 40 °C and 60% relative humidity and subjected to solar light radiation (1.2 W/m^2^) emitted by a lamp supplied with the chamber. Samples were then subjected to static contact angle measurement and to capillary water absorption testing in order to evaluate the performance of treatments after artificial ageing. 

## Figures and Tables

**Table 1:** Results of XRD analysis (+++: dominantly present, ++: present, +: traces) and calcite and dolomite content (%) measured by the Dietrich–Frühling gas volumetric method.

Substrate	XRD				Dietrich–Frühling Method	
	Calcite	Quartz	Fluorapatite	Dolomite	Calcite (%)	Dolomite (%)
sandstone	+++	++	-	-	88	-
limestone	+++	-	+	-	86	-
marble	+++	-	-	+	98	2

**Table 2:** Sample codes.

Stone	Polymer	Dip Coating	Poultice Coating	Spray Coating
sandstone	PHB	D-SANDs-PHB	P-SANDs-PHB	S-SANDs-PHB
	PHBVV	D-SANDs-PHBVV	P-SANDs-PHBVV	S-SANDs-PHBVV
	Sol-SIL	D-SANDs-Sol-SIL	P-SANDs-Sol-SIL	S-SANDs-Sol-SIL
	Emul-SIL	D-SANDs-Emul-SIL	P-SANDs-Emul-SIL	S-SANDs-Emul-SIL
limestone	PHB	D-LIMEs-PHB	P-LIMEs-PHB	S-LIMEs-PHB
	PHBVV	D-LIMEs-PHBVV	P-LIMEs-PHBVV	S-LIMEs-PHBVV
	Sol-SIL	D-LIMEs-Sol-SIL	P-LIMEs-Sol-SIL	S-LIMEs-Sol-SIL
	Emul-SIL	D-LIMEs-Emul-SIL	P-LIMEs-Emul-SIL	S-LIMEs-Emul-SIL
marble	PHB	D-MARBLE-PHB	P-MARBLE-PHB	S-MARBLE-PHB
	PHBVV	D-MARBLE-PHBVV	P-MARBLE-PHBVV	S-MARBLE-PHBVV
	Sol-SIL	D-MARBLE-Sol-SIL	P-MARBLE-Sol-SIL	S-MARBLE-Sol-SIL
	Emul-SIL	D-MARBLE-Emul-SIL	P-MARBLE-Emul-SIL	S-MARBLE-Emul-SIL

**Table 3:** Determination of the mean ratio of protection (*Rp*, %) for treated samples of sandstone after 1 h and after 48 h of capillary absorption test.

Application Method	Sandstone							
	PHB		PHBVV		Sol-SIL		Emul-SIL	
	Rp (%) after 1 h	Rp (%) after 48 h	Rp (%) after 1 h	Rp (%) after 48 h	Rp (%) after 1 h	Rp (%) after 48 h	Rp (%) after 1 h	Rp (%) after 48 h
dip coating	94	84	92	86	98	92	96	97
poultice	100	90	96	92	n.a.	n.a.	n.a.	n.a.
spray	85	75	92	90	90	89	91	90

**Table 4:** Determination of the mean ratio of protection (Rp, %) for treated samples of sandstone after 1 h and after 48 h of capillary absorption test.

Application Method	Limestone							
	PHB		PHBVV		Sol-SIL		Emul-SIL	
	Rp (%) after 1 h	Rp (%) after 48 h	Rp (%) after 1 h	Rp (%) after 48 h	Rp (%) after 1 h	Rp (%) after 48 h	Rp (%) after 1 h	Rp (%) after 48 h
dip coating	0	0	99	96	99	95	99	87
poultice	98	43	98	94	n.a.	n.a.	n.a.	n.a.
spray	68	28	76	91	98	95	98	88

**Table 5:** Static contact angle (*θ*) and dynamic contact angles referred to the first immersion cycle (*θ*_adv1_, *θ*_rec1_) for untreated and treated sandstone.

SAMPLE	Static Contact Angle	Dynamic Contact Angle	
	*θ* (°)	*θ*_adv1_ (°)	*θ*_rec1_ (°)
UT-SANDs	15 ± 4	40	0
D-SANDs-PHB	95 ± 8	104	0
P-SANDs-PHB	93 ± 9	113	19
S-SANDs-PHB	97 ± 11	102	8
D-SANDs-PHBVV	104 ± 12	113	0
P-SANDs-PHBVV	123 ± 9	126	17
S-SANDs-PHBVV	101 ± 8	107	0
D-SANDs-Sol-SIL	140 ± 1	146	33
S-SANDs-Sol-SIL	142 ± 4	134	25
D-SANDs-Emul-SIL	125 ± 4	132	28
S-SANDs-Emul-SIL	124 ± 1	138	26

**Table 6:** Static contact angle (*θ*) and dynamic contact angles referred to the first immersion cycle (*θ*_adv1_, *θ*_rec1_) for untreated and treated limestone.

SAMPLE	Static Contact Angle	Dynamic Contact Angle	
	*θ* (°)	*θ*_adv1_ (°)	*θ*_rec1_ (°)
UT-LIMEs	0 ± 0	16	0
D-LIMEs-PHB	108 ± 7	119	0
P-LIMEs-PHB	112 ± 5	110	0
S-LIMEs-PHB	113 ± 6	124	0
D-LIMEs-PHBVV	119 ± 4	128	26
P-LIMEs-PHBVV	126 ± 7	117	25
S-LIMEs-PHBVV	120 ± 6	122	0
D-LIMEs-Sol-SIL	143 ± 2	143	38
S-LIMEs-Sol-SIL	146 ± 1	166	48
D-LIMEs-Emul-SIL	118 ± 1	150	0
S-LIMEs-Emul-SIL	124 ± 1	141	0

**Table 7:** Static contact angle (*θ*) and dynamic contact angles referred to the first immersion cycle (*θ*_adv1_, *θ*_rec1_) for untreated and treated marble.

SAMPLE	Static Contact Angle	Dynamic Contact Angle	
	*θ* (°)	*θ*_adv1_ (°)	*θ*_rec1_ (°)
UT-Marble	41 ± 7	60	19
D-Marble-PHB	80 ± 6	78	29
P-Marble-PHB	80 ± 9	92	26
S-Marble-PHB	79 ± 6	102	8
D-Marble-PHBVV	84 ± 4	85	30
P-Marble-PHBVV	109 ± 10	104	24
S-Marble-PHBVV	84 ± 4	92	41
D-Marble-Sol-SIL	120 ± 6	133	17
D-Marble-Emul-SIL	119 ± 4	108	34

**Table 8:** Average ΔL*, Δa* and Δb* values of sandstone, limestone and marble treated with PHB and PHBVV with respect to untreated conditions.

Colour Coordinate	Sandstone		Limestone		Marble	
	PHB	PHBVV	PHB	PHBVV	PHB	PHBVV
ΔL*	2.5 ± 1.5	2.5 ± 1.5	1.7 ± 2.5	4.1 ± 2.7	0.8 ± 0.4	0.8 ± 0.6
Δa*	−0.4 ± 0.2	−0.4 ± 0.4	−0.6 ± 0.3	−0.5 ± 0.2	0.1 ± 0.1	0.1 ± 0.1
Δb*	−2 ± 1.6	−2.3 ± 2.5	−3.6 ± 0.9	−3.2 ± 2.3	0.3 ± 0.4	−0.4 ± 0.2

**Table 9:** Results of water vapour diffusion test for sandstone treated with PHB and PHBVV and untreated.

Sample	*V*_S_ or *V*_CS_ (g/m^2^·Day)	*V* (g/m^2^·Day)	*S*_d_ (m)	Water Vapour Transmission Rate Class
UT-SANDs	*V*_S_ = 86	-	-	-
D-SANDs-PHB	*V*_CS_ = 67	302	0.10	high
P-SANDs-PHB	*V*_CS_ = 93	>680	0.03	high
S-SANDs-PHB	*V*_CS_ = 59	190	0.16	medium
D-SANDs-PHBVV	*V*_CS_ = 60	194	0.16	medium
P-SANDs-PHBVV	*V*_CS_ = 76	>680	0.05	high
S-SANDs-PHBVV	*V*_CS_ = 80	>680	0.03	high

**Table 10:** Results of water vapour diffusion test for limestone treated with PHB and PHBVV and untreated.

Sample	*V*_S_ or *V*_CS_ (g/m^2^·Day)	*V* (g/m^2^·Day)	*S*_d_ (m)	Water Vapour Transmission Rate Class
UT-LIMEs	*V*_S_ = 278	-	-	-
D-LIMEs-PHB	*V*_CS_ = 306	>680	0.01	high
P-LIMEs-PHB	*V*_CS_ = 266	>680	0.01	high
S-LIMEs-PHB	*V*_CS_ = 163	391	0.08	high
D-LIMEs-PHBVV	*V*_CS_ = 126	231	0.14	high
P-LIMEs-PHBVV	*V*_CS_ = 326	>680	0.02	high
S-LIMEs-PHBVV	*V*_CS_ = 295	>680	0.01	high

**Table 11:** Results of water vapour diffusion test for marble treated with PHB and PHBVV and untreated.

Sample	*V*_S_ or *V*_CS_ (g/m^2^·Day)	*V* (g/m^2^·Day)	*S*_d_ (m)	Water Vapour Transmission Rate Class
UT-MARBLE	*V*_S_ = 21	-	-	-
D-MARBLE-PHB	*V*_CS_ = 24	171	0.18	medium
P-MARBLE-PHB	*V*_CS_ = 15	49	0.64	medium
S-MARBLE-PHB	*V*_CS_ = 11	24	1.32	medium
D-MARBLE-PHBVV	*V*_CS_ = 25	124	0.25	medium
P-MARBLE-PHBVV	*V*_CS_ = 23	199	0.16	medium
S-MARBLE-PHBVV	*V*_CS_ =18	130	0.24	medium

**Table 12:** Static contact angle values determined after the artificial ageing (c.a.: complete absorption; n.d.: not determined).

Protective	Application Method	Sandstone	Limestone	Marble
		*θ* (°)	*θ* (°)	*θ* (°)
PHB	dip coating	6 ± 6	c.a.	21 ± 6
	poultice	21 ± 7	c.a.	21 ± 7
	spray	17 ± 0	c.a.	17 ± 5
PHBVV	dip coating	14 ± 3	c.a.	39 ± 8
	poultice	14 ± 4	c.a.	21 ± 12
	spray	16 ± 3	c.a.	26 ± 8
Sol-SIL	dip coating	123 ± 5	128 ± 3	112 ± 8
	spray	126 ± 7	125 ± 4	n.d.
Emul-SIL	dip coating	123 ± 6	127 ± 4	110 ± 9
	spray	124 ± 6	129 ± 5	n.d.

**Table 13:** Mean ratio of protection of the treatments on sandstone after the artificial ageing (n.d.: not determined).

Application Method	PHB		PHBVV		Sol-SIL		Emul-SIL	
	Rp (%) after 1 h	Rp (%) after 48 h	Rp (%) after 1 h	Rp (%) after 48 h	Rp (%) after 1 h	Rp (%) after 48 h	Rp (%) after 1 h	Rp (%) after 48 h
dip coating	80	71	77	54	91	90	94	88
poultice	83	80	83	85	n.d.	n.d.	n.d.	n.d.
spray	0	0	74	66	91	83	100	95

**Table 14:** Mean ratio of protection of the treatments on limestone after the artificial ageing (n.d.: not determined).

Application Method	PHB		PHBVV		Sol-SIL		Emul-SIL	
	Rp (%) after 1 h	Rp (%) after 48 h	Rp (%) after 1 h	Rp (%) after 48 h	Rp (%) after 1 h	Rp (%) after 48 h	Rp (%) after 1 h	Rp (%) after 48 h
dip coating	0	6	0	34	98	96	96	90
poultice	45	32	41	27	n.d.	n.d.	n.d.	n.d.
spray	25	46	82	19	98	97	92	80
