# Erratum: Andreotti, S.; Franzoni, E.; Fabbri, P. Poly(hydroxyalkanoate)s-Based Hydrophobic Coatings for the Protection of Stone in Cultural Heritage. *Materials* 2018, *11*, 165

**DOI:** 10.3390/ma11030389

**Published:** 2018-03-07

**Authors:** Serena Andreotti, Elisa Franzoni, Micaela Degli Esposti, Paola Fabbri

**Affiliations:** 1 Department of Civil, Chemical, Environmental and Materials Engineering, University of Bologna, 40131 Bologna, Italy; serena.andreotti2@unibo.it (S.A.); micaela.degliesposti@unibo.it (M.D.E.); p.fabbri@unibo.it (P.F.); 2 Consorzio Interuniversitario di Scienza e Tecnologia dei Materiali (INSTM), 50121 Firenze, Italy

The authors wish to add a new author, Micaela Degli Esposti, who also contributed to performing the experiments and analyzing the data of this published paper [1]. The correct authorship is shown below:

Serena Andreotti ^1,2^, Elisa Franzoni ^1,2,^*, Micaela Degli Esposti ^1,2^ and Paola Fabbri ^1,2^

^1^ Department of Civil, Chemical, Environmental and Materials Engineering, University of Bologna, 40131 Bologna, Italy; serena.andreotti2@unibo.it (S.A.); micaela.degliesposti@unibo.it (M.D.E.); p.fabbri@unibo.it (P.F.)

^2^ Consorzio Interuniversitario di Scienza e Tecnologia dei Materiali (INSTM), 50121 Firenze, Italy

* Correspondence: elisa.franzoni@unibo.it; Tel.: +39-051-209-0339
